# Confirmatory detection and identification of biotic and abiotic stresses in wheat using Raman spectroscopy

**DOI:** 10.3389/fpls.2022.1035522

**Published:** 2022-10-17

**Authors:** Samantha Higgins, Valeryia Serada, Benjamin Herron, Kiran R. Gadhave, Dmitry Kurouski

**Affiliations:** ^1^ Department of Biochemistry and Biophysics, Texas A&M University, College Station, TX, United States; ^2^ Texas A&M AgriLife Research, Amarillo, TX, United States; ^3^ Department of Entomology, Texas A&M University, College Station, TX, United States

**Keywords:** wheat, Raman spectroscopy, PLS-DA, biotic stress, abiotic stress

## Abstract

Wheat is one of the oldest and most widely cultivated staple food crops worldwide. Wheat encounters an array of biotic and abiotic stresses during its growth that significantly impact the crop yield and consequently global food security. Molecular and imaging methods that can be used to detect such stresses are laborious and have numerous limitations. This catalyzes the search for alternative techniques that can be used to monitor plant health. Raman spectroscopy (RS) is a modern analytical technique that is capable of probing structure and composition of samples non-invasively and non-destructively. In this study, we investigate the accuracy of RS in confirmatory diagnostics of biotic and abiotic stresses in wheat. Specifically, we modelled nitrogen deficiency (ND) and drought, key abiotic stresses, and Russian wheat aphid (Diuraphis noxia) infestation and viral diseases: wheat streak mosaic virus (WSMV) and Triticum mosaic virus (TriMV), economically significant biotic stresses in common bread wheat. Raman spectra as well as high pressure liquid chromatography (HPLC)-based analyses revealed drastically distinct changes in the intensity of carotenoid vibration (1185 cm^-1^) and in the concentration of lutein, chlorophyll, and pheophytin biomolecules of wheat, triggered in response to aforementioned biotic and abiotic stresses. The biochemical changes were reflected in unique vibrational signatures in the corresponding Raman spectra, which, in turn could be used for ~100% accurate identification of biotic and abiotic stresses in wheat. These results demonstrate that a hand-held Raman spectrometer could provide an efficient, scalable, and accurate diagnosis of both biotic as well as abiotic stresses in the field.

## Introduction

Wheat (Triticum aestivum) is one of the most important and broadly cultivated crops in the world. In 2017 alone, wheat production reached 750 million tons (Food and Agriculture Organization of the United Nations, 2009). This crop is cultivated in 124 different countries accounting for over 18% of the total food supply of the world (Freije et al., 2016).

Global food security can be quickly jeopardized by several biotic and abiotic stresses that staple food crops, such as wheat, encounter during critical growth stages. For instance, crop yield directly depends on primary, secondary and micronutrients in soil. Nitrogen, arguably the most consequential element for plant growth, is predominantly utilized by plants to synthesize chlorophyll, an important photosynthetic pigment (Ding et al., 2005; Pandey et al., 2017). Upon nitrogen deficiency (ND), wheat growth decelerates, and plant leaves exhibit chlorosis. Morphologically similar symptoms are observed in wheat upon drought stress that drastically reduces crop yield. In turn, morphological appearance of both ND and drought is very similar to viral diseases caused by wheat streak mosaic virus (WSMV) and Triticum mosaic virus (TriMV) (Mascia and Gallitelli, 2016; Bryan et al., 2019). These viruses are vectored by the wheat curl mites. Both WSMV and BYDV are filamentous ssRNA viruses of the Potyviridae family that cause devastating damages in various parts of the world, including Great Plains region of the United States (Seifers et al., 2008).

Confirmatory diagnostics of WSMV and TriMV can be achieved using polymerase chain reaction (PCR) or protein based analyses (Martinelli et al., 2014). Although accurate, both analyses are laborious and require sample shipment that increases direct costs of diagnostics. Morphological similarities of biotic (viral) and abiotic (drought and ND) stresses further lowers the efficiency of PCR- and qPCR, as these molecular methods of analyses fail to detect abiotic stresses. ND can be determined by nitrate extraction from plant samples using a 1 M KCl solution (Sáez-Plaza et al., 2013). After nitrate reduction to nitrite, the concentration of nitrites can then be determined by spectrophotometric measurement such as high temperature combustion, atomic absorption spectroscopy, and atomic absorption spectrophotometry (ICP) (Zaman et al., 2018). These analytical approaches are laborious and destructive, as well as require sample shipment to analytical laboratories. Unlike ND, there is no reliable analytical method that allows for confirmation of drought stress in plants. Imaging methods, including thermography, hyperspectral, and RGB, can be used to detect changes in the color, texture, or temperature of plants (Baena et al., 2017). However, utilization of such imaging methods for detection of drought stress is problematic because visually drought stress is highly similar to ND and viral diseases (Waraich et al., 2012; Ding et al., 2005). These limitations of currently available molecular and imaging techniques catalyzed the search for an approach that can be used for confirmatory identification of biotic and abiotic stresses in wheat.

A growing body of evidence suggests that Raman spectroscopy (RS), an emerging analytical technique, can be used for detection of plant stresses. Sanchez and co-workers previously demonstrated that RS could be used to detect nitrogen, phosphorus, and potassium deficiencies, as well as salinity stress in rice (Yeturu et al., 2016; Egging et al., 2018; Farber and Kurouski, 2018; Mandrile et al., 2019; Sanchez et al., 2019a; Sanchez et al., 2019c; Huang et al., 2020; Sanchez et al., 2020b; Sanchez et al., 2020c). Furthermore, RS could be used to identify fungal pathogen on species level in wheat, sorghum, and corn (Egging et al., 2018; Farber and Kurouski, 2018). Morey and co-workers recently discovered that RS could be used to detect drought and salinity stresses in peanuts. Although RS is generally known to be a laboratory-based method, the past decade has seen several developments of portable Raman spectrometers. These hand-held instruments can be utilized directly in the field (Yeturu et al., 2016; Sanchez et al., 2019b; Sanchez et al., 2019c; Sanchez et al., 2020a). This technological development sparked the interest of agronomists, plant pathologists and plant biologists in utilization of this technology for analysis of the plant health status.

In this study, we examined the potential of RS in confirmatory identification of abiotic (drought and ND), as well as biotic (Russian wheat aphid and mixed viral infection of WSMV+TriMV) stresses in wheat. To achieve this, we modeled these biotic and abiotic stresses in the greenhouse and analyzed plant leaves using RS. Furthermore, we coupled RS to chemometric analysis to determine the accuracy of Raman-based identification of biotic and abiotic stresses in plants. In parallel, we performed analysis of metabolic changes in plants that took place upon drought, ND, aphid-induced stress and bi-viral WSMV+TriMV infection using high performance liquid chromatography (HPLC). Our findings show that these stresses caused drastic changes in the carotenoid profiles of wheat. Similar changes in intensities of vibrational bands that originate from carotenoids were observed in the corresponding Raman spectra. These findings provide a proof-of-concept data on changes in plant carotenoids, which enables confirmatory diagnostics of biotic and abiotic stresses in wheat.

## Methods

### Plants

Wheat (cv. TAM304) seeds were planted in trays (6 x 5 cells, 4.2 in3/cell/plant) which were placed in W60 x D60 x H60 cm insect-proof cages (MegaView Science Co., Taichung, Taiwan) (one tray/cage/treatment) and maintained in an automated greenhouse under controlled environmental conditions (25°C temperature, 14-hr photoperiod) in Bushland, TX. Plants were arranged in five treatments: healthy control, water-stressed (i.e., drought), ND, aphid-stressed and disease-stressed (WSMV+TriMV) with 30 replicates per treatment to ensure plant survival. Seeds from nitrogen deficient treatment were planted in sand, whereas in all other treatments, planted in Sungro professional growing mix (Sungro Horticulture). Plants across all treatments were watered as required (every 1-2 days), except drought treatment in which plants were watered every four days with bare minimal quantity required for wheat survival (based on an initial assessment) and ND treatment, in which plants were watered daily to ensure plant survival because of the poor water holding capacity of sand. Two and half weeks after planting, 20 adults of Russian wheat aphid (colony maintained in the Entomology greenhouse at Bushland, TX) were evenly distributed among 30 replicate plants in the aphid-stressed treatment. At the same time, 20 1-cm leaf segments infested with WSMV and TriMV viruliferous wheat curl mites were evenly distributed among 30 replicate plants in the disease-stressed treatment. Plants from all treatments were cut at ground level seven weeks after planting when both aphid infestation and disease incidence peaked. All samples were shipped overnight on ice in a Styrofoam container to College Station, TX for subsequent analysis. The presence of both viruses in the disease treatment was confirmed using one-step qRT-PCR (Tatineni et al., 2010).

### Raman spectroscopy

**Figure 1:**
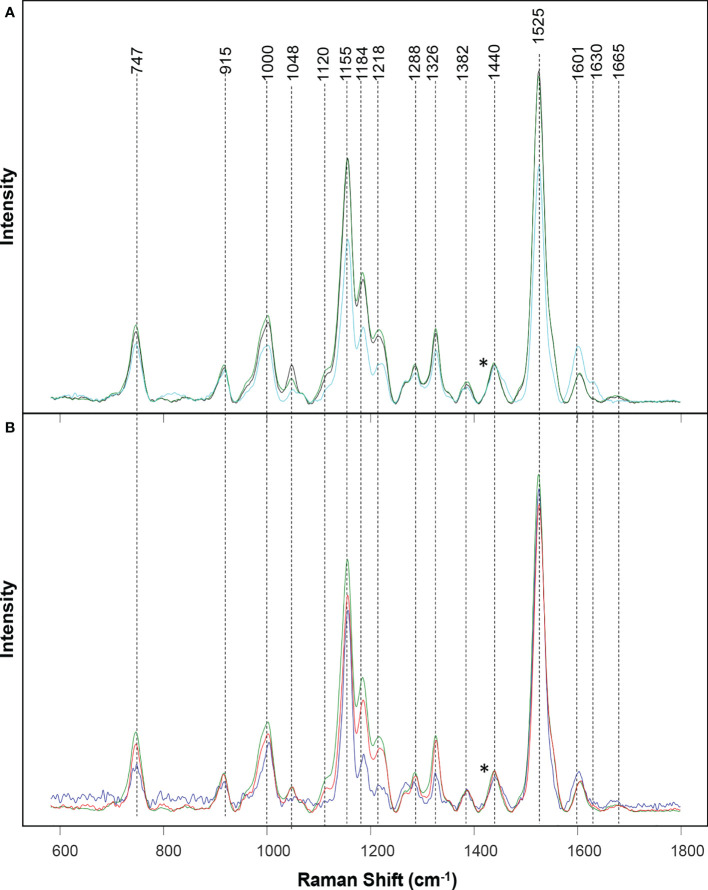
Raman spectra collected from leaves of healthy (green), ND- (light blue) and drought (black) stressed plants **(A)**, as well as wheat exposed to WSMV+TriMV infection (red) and aphid stress (blue) **(B)**. *Spectra normalized on 1,440 cm^-1^ vibrational band, which were assigned to CH2 vibration.

Raman spectra of wheat leaves were measured with a hand-held Resolve Agilent spectrometer with 831 nm laser. The following experimental parameters were used for all collected spectra: 1s acquisition time, 495 mW power, and baseline spectral subtraction by device software. We recorded four spectra from each leaf at four quadrants on its adaxial side. In total, around 50 surface spectra were recorded from each stress type, as well as healthy plants. Spectra shown in the **Figure 1** are raw baseline corrected, without smoothing.

### Data analysis

MATLAB R2020a (Mathworks) equipped with PLS_Toolbox (Eigenvector Research Inc.) was used for all data analysis. First, the spectra were normalized at the 1440 cm^-1^ band. Second, the spectra were baselined using automatic weighted least squares to the second order then the first derivative was taken of the Raman spectra with a filter width of 15 and polynomial order 2. Third, the spectra were area normalized then multiplicative signal correction based on the mean was applied to all spectra. A partial least squares discriminant analysis (PLS-DA) was performed for all classes of spectra presented in the results and discussion of this manuscript. The imported spectra wavenumbers that were analyzed were included from 300 cm^-1^ to 1765 cm^-1^ which includes all important spectra characteristics of wheat.

Analysis of variance (ANOVA) was used to determine the changes at observed bands. The null hypothesis of this test is that there is no significant difference at the band of interests. A significant level (α) is 0.05. The ANOVA also reported a 95% confidence interval for the true value of median for each compared group, **Figure S1**. The overlapping confidence intervals were conducted using MATLAB multcompare function, which by default uses Tukey HSD to evaluate group-to-group differences.

### Carotenoid extraction

Wheat leaf samples (~150 mg) were homogenized using a mortar and pestle. After, a 1.5 mL solution of chloroform and dichloromethane (2:1, v/v) was added to the homogenate, the mixture was agitated on thermomixer at 500 rpm at 4°C for 30 minutes. To achieve a phase separation, 0.5 mL of 1 M sodium chloride solution was added to the homogenate and mixed by inversion. Next, the solution was centrifuged at 5,000 g for 10 minutes. The aqueous and organic phases were separated in different tubes. The aqueous phase was subjected to another round of separation by adding 0.75 mL of chloroform and dichloromethane (2:1, v/v), followed by centrifugation at 5,000 g for 10 minutes. The second organic phase was collected and pooled with the first batch and dried by centrifugal evaporation method. Dried pellet was re-dissolved in 1mL of methanol/tert-methyl butyl ether (MTBE) (60/40, v/v) prior to injection into HPLC.

### HPLC analysis

Leaf extracts were analyzed by reversed phase HPLC using Waters 1525 pump equipped with Waters 2707 auto sampler and 2489 Waters photodiode array detector (PDA). Carotenoids were separated on a reverse-phase C30, 3 μm column (250 × 4.6 mm) (Thermo Fisher Scientific Inc, part number 075723) using mobile phases consisting of (A) methanol/water (95:5, v/v) and (B) MTBE. The gradient elution used with this column was 97% A and 3% B at 0-6 min with a linear increase of B to 100% at 20 min and return to initial conditions at 23 min. The column temperature was maintained at 20°C. The eluting peaks were monitored at 450 nm using PDA. Quantification was performed using Breeze software comparing peak area with standard reference curves.

## Results and discussion

Raman spectra acquired from healthy wheat leaves exhibit vibrational bands that can be assigned to carbohydrates (747 and 915 cm^-1^), carotenoids (1000, 1048, 1120, 1155, 1184, 1218, and 1525 cm^-1^), polyphenols (1601 and 1630 cm^-1^), as well as proteins (1665 cm^-1^) **Table 1**. We also observed vibrational bands that can be assigned to CH and CH2 vibrations (1288, 1326, 1382 and 1440 cm^-1^). These chemical groups are typically present in nearly all classes of biological molecules. Therefore, these bands cannot be assigned to a particular class of chemical compounds specified above.

**Table 1:** Vibrational band assignments for wheat leaf spectra.

Band	Vibrational mode	Assignment
747	γ(C–O-H) of COOH	pectin (Synytsya et al., 2003)
915	ν(C-O-C) in plane, symmetric	cellulose, lignin (Edwards et al., 1997)
1000	in-plane CH_3_ rocking of polyene	carotenoids (Adar, 2017; Devitt et al., 2018), proteins (Devitt et al., 2018)
1048	-C=C-	carotenoids (Adar, 2017; Devitt et al., 2018)
1120	-C=C-	carotenoids (Adar, 2017; Devitt et al., 2018)
1155	-C=C-	carotenoids (Adar, 2017; Devitt et al., 2018)
1184	-C=C-	carotenoids (Adar, 2017; Devitt et al., 2018)
1218	-C=C-	carotenoids (Adar, 2017; Devitt et al., 2018)
1288	δ(C-C-H)	aliphatics (Yu et al., 2007)
1326	δCH_2_ bending vibration	cellulose, lignin (Edwards et al., 1997)
1382	δCH_2_ bending vibration	aliphatics (Yu et al., 2007)
1440	δ(CH_2_)+δ(CH_3_)	aliphatics (Yu et al., 2007)
1525	-C=C- (in plane)	carotenoids (Adar, 2017; Devitt et al., 2018)
1601	ν(C-C) aromatic ring+σ(CH)	lignin (Agarwal, 2006; Kang et al., 2016)
1630	C=C-C (ring)	lignin (Agarwal, 2006; Kang et al., 2016; Pompeu et al., 2017)
1665	C=O stretching, amide I	proteins (Devitt et al., 2018)

Similar vibrational bands were observed in the spectra collected from wheat exposed to each different type of stress. For instance, in the Raman spectra collected from wheat exposed to drought, we observed a small decrease in the intensity of carotenoid (1000, 1048, 1120, 1155, 1184, 1218, and 1525 cm^-1^) bands, **Figure 1** and **Table S1**. These observations suggest that drought causes a decrease in the concentration of carotenoids in plants, thereby showing harmony with earlier findings by Morey and co-workers (Morey et al., 2021). We observed even greater decrease in the intensity of carotenoid vibrations in the spectra collected from ND plants. Furthermore, we found an increase in the intensity of polyphenol (1601 and 1630 cm^-1^) vibrations, as well as a decrease in the intensity of 1665 cm^-1^. These spectral changes point to drastically different changes in plant biochemistry that are taken place upon ND compared to the drought stress. Specifically, in addition to the decrease in the concentration of carotenoids, ND is associated with an increase in the concentration of phenylpropanoids and a decrease in the concentration of proteins in plants. These results are in a good agreement with the previously reported findings by Sanchez and co-workers (Sanchez et al., 2020b).

We also observed a decrease in the intensity of carotenoid vibrations in the Raman spectra collected from wheat leaves mixed infected with WSMV+TriMV and those infested with aphids. It is important to note that a decrease in the intensity of carotenoids was much greater in the spectrum collected from aphid-stressed wheat compared to plants experienced viral infection. Furthermore, we observed a small shift in the polyphenol vibration from 1601 cm^-1^ (healthy wheat) to 1598 cm^-1^ in the spectra collected from aphid-stressed wheat. These results suggest that biotic and abiotic stresses cause distinctly different changes in plant biochemistry, which, in turn, can be detected by RS to enable confirmatory, non-invasive, and non-destructive diagnostics of plant health.

Previously reported studies by our and other research groups point to the drastic changes in biochemistry of carotenoids upon biotic stresses caused by bacteria and viruses. A decrease in the carotenoid content upon such stresses has strong physiological relevance (Havaux, 2013). Plant defense mechanism is based on activation of enzymatic oxidation of carotenoids that results in formation of abscisic acid, a hormone that enhances plant resistance to such stresses (Nambara and Marion-Poll, 2005). Furthermore, oxidation and cleavage of β-carotene by reactive oxygen species (ROS) yields β-lonone, β-cyclocitrals that can protect the plant against insects (Nambara and Marion-Poll, 2005; Havaux, 2013). Thus, reduction in this molecule could be a consequence of the higher ROS commonly triggered during plant defense reactions (Yu et al., 2007).

**Figure 2:**
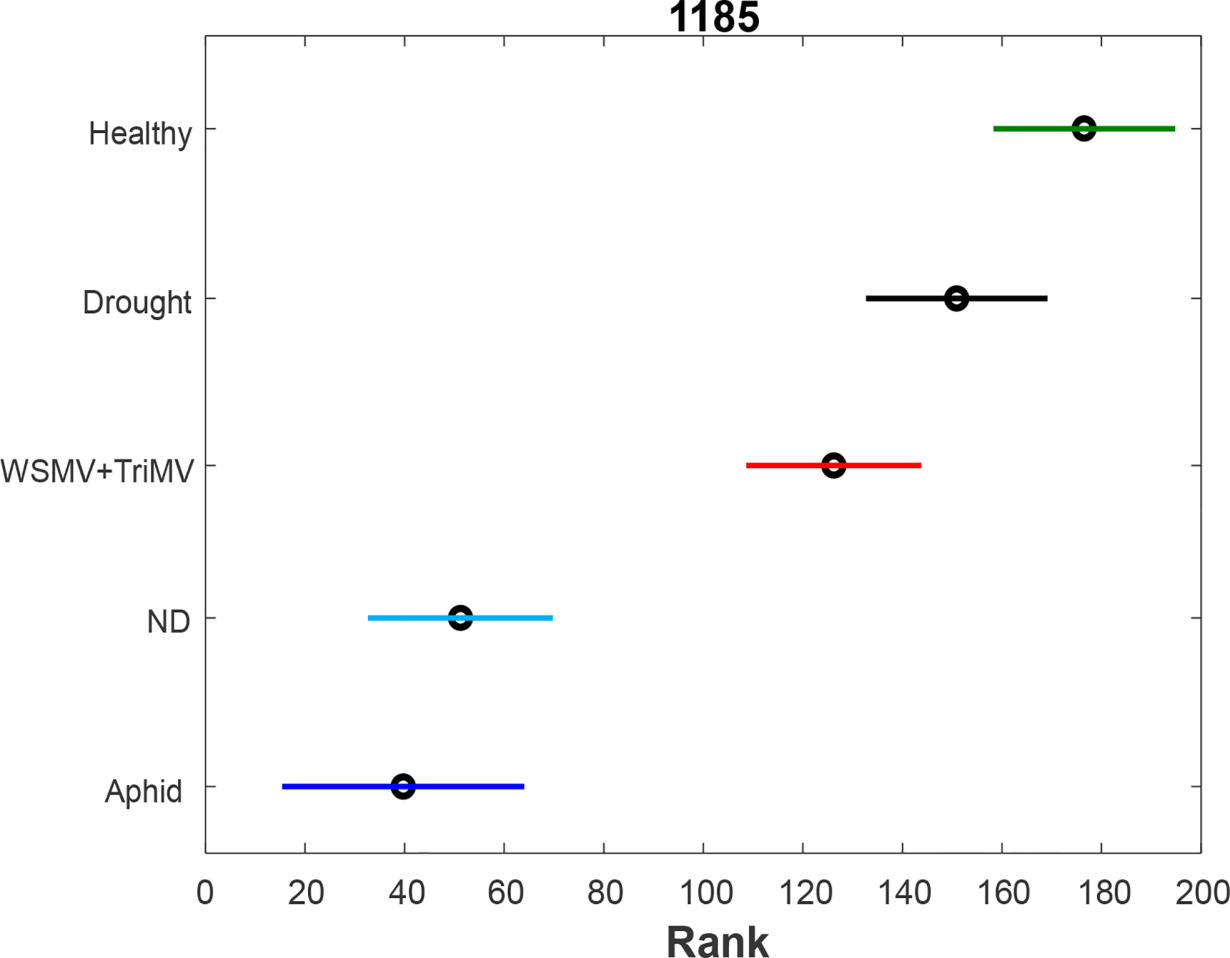
ANOVA (A) of carotenoid vibration (1185 cm-1) in the spectra of healthy (green), ND- (light blue) and drought (black) stressed plants, as well as wheat exposed to WSMV+TriMV infection (red) and aphid stress (blue).

To further investigate changes in carotenoid biochemistry in response to biotic and abiotic stresses, we compared intensity of carotenoid vibration (1185 cm^-1^) in Raman spectra collected from wheat leaves, **Figures 2**, **S1**. PLS-DA results show that different biotic and abiotic stresses can be accurately predicted based on the corresponding Raman spectra, **Table 2**. Specifically, the model highly accurately predicted ND, WSMV+TriMV infection and aphid stress (100%), whereas very good identification of drought (96%) and healthy plants (control, 98%) could be achieved.

**Table 2:** Accuracy of classification by PLS-DA wild plant species.

	Accuracy, %	Aphid	Drought	Healthy	ND	WSMV+TriMV
Aphid	100	29	0	0	0	0
Drought	96	0	48	1	0	0
Healthy	98	0	2	49	0	0
ND	100	0	0	0	48	0
WSMV+TriMV	100	0	0	0	0	54

We found that all biotic and abiotic stresses exhibit a decrease in the concentration of carotenoids. Furthermore, such changes are greater for ND and aphid stress compared to WSMV+TriMV infection. Changes of carotenoids upon drought stress are non-significant compared to healthy plants.

**Figure 3:**
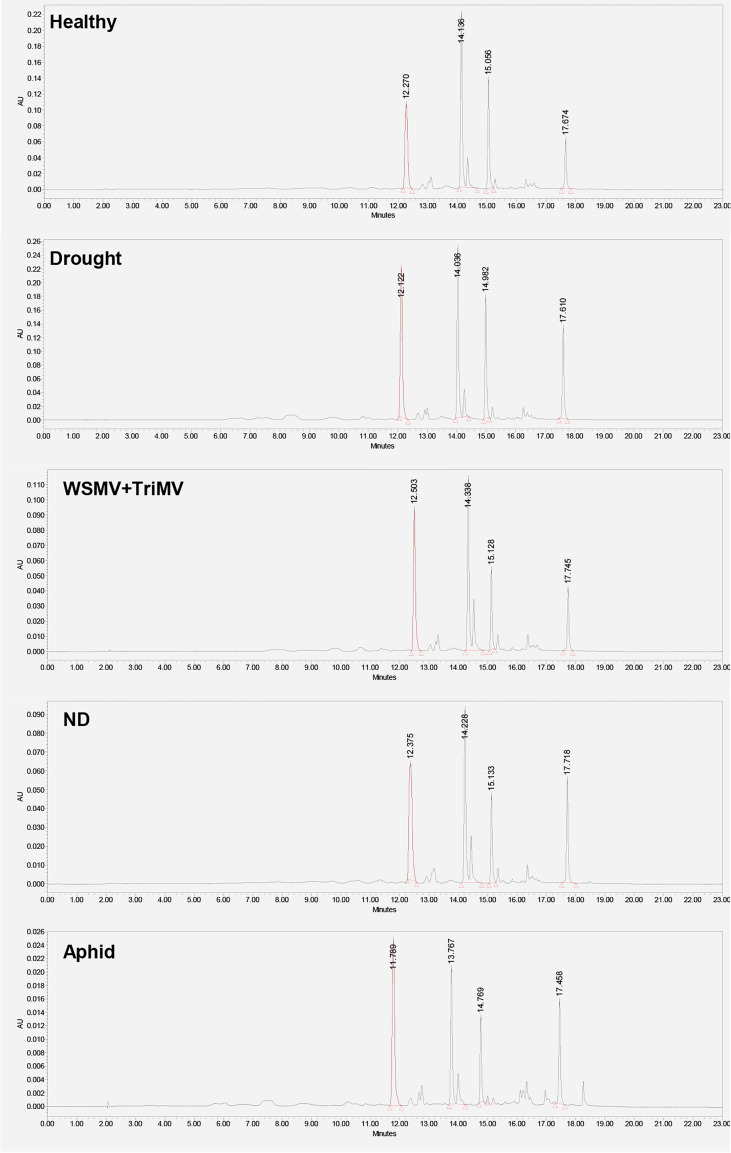
HPLC profiles of leaves of healthy wheat, as well as wheat exposed to drought, WSMV+TriMV infection, ND and aphid stresses.

**Figure 4:**
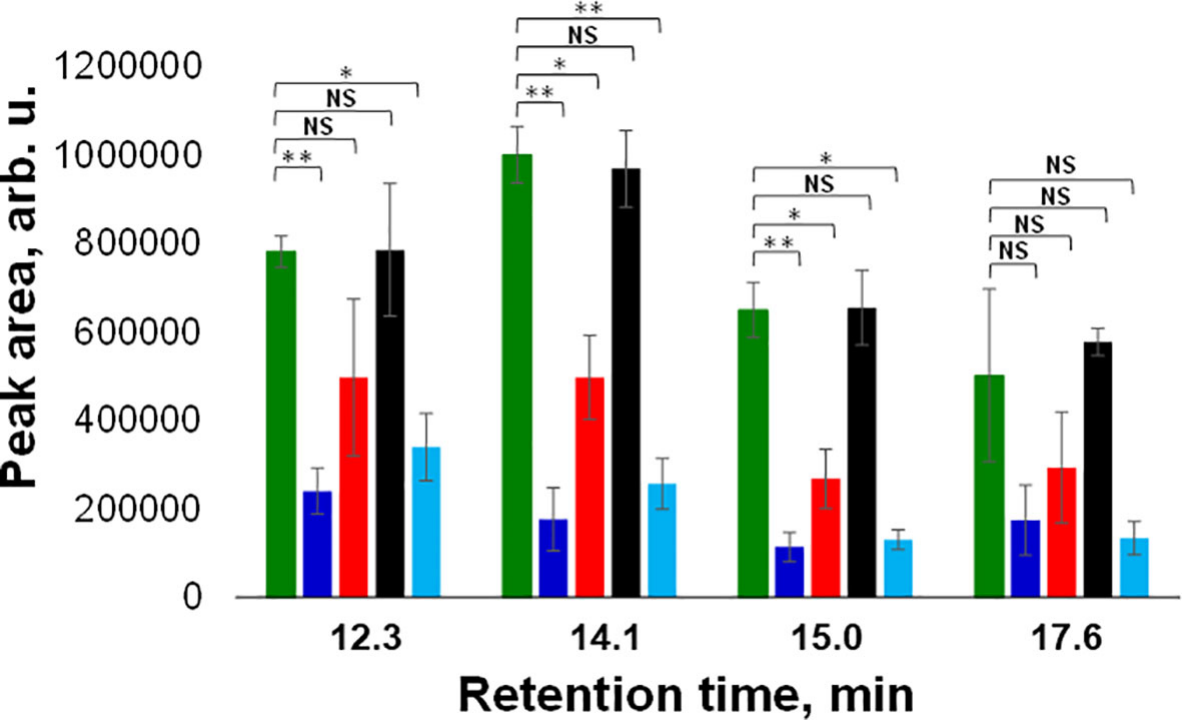
Average peak area and corresponding standard deviations of peaks observed in triplicates of HPLC profiles of healthy (green), ND- (light blue) and drought (black) stressed plants, as well as wheat exposed to WSMV+TriMV infection (red) and aphid stress (blue). RT=12.3 min = lutein; RT=14.1 min = chlorophyll; RT=15.0 min = pheophytin; RT=17.6 min = b-carotene. NS is a nonsignificant difference, and *P ≤ 0.05, **P ≤ 0.01.

Next, we utilized HPLC to identify changes in carotenoid profile of wheat. We found that HPLC profile of healthy wheat was dominated by 4 carotenoids: lutein (RT=12.3 min), chlorophyll (RT=14.1 min), pheophytin (RT=15.0 min) and β-carotene (RT=17.6 min), **Figure 3**. Peaks with the same or similar RTs were observed in HPLC profiles of wheat exposed to drought, WSMV+TriMV infection, ND, and aphid stress. It should be noted that we observed appearance of a new peak with RT=18.4 min in the HPLC profile of aphid wheat, **Figure 3**. However, we observed changes in the intensities of lutein (RT=12.3 min), chlorophyll (RT=14.1 min), pheophytin (RT=15.0 min) and β-carotene (RT=17.6 min) peaks in the HPLC profiles of wheat with biotic and abiotic stresses, **Figure 4**.

Specifically, we observed a significant decrease in the concentration of lutein in ND and aphid-stressed plants compared to the concentration of lutein in healthy wheat. We also found significant decrease in the concentrations of chlorophyll and pheophytin in ND, WSMV+TriMV infected and aphid-stressed wheat. Finally, no statistically significant changes were observed in the concertation of β-carotene in wheat exposed to any of the biotic or abiotic stress relative to the concentration of this carotenoids in the control plants. These results demonstrate that WSMV+TriMV infection, ND and aphid stresses caused drastic decrease in the concentration of lutein, chlorophyll and pheophytin in wheat. Furthermore, a decrease in the concentration of these carotenoids matches with the changes in the 1185 cm^-1^ band in the Raman spectra acquired from plants exposed to biotic and abiotic stresses. Our group previously demonstrated that chlorophyll and pheophytin are highly fluorescent and provide no Raman spectra at 830 nm excitation. Thus, we can conclude that RS detects changes in lutein in wheat, which enables confirmatory detection and identification of plant biotic and abiotic stresses.

## Conclusions

Our results show that RS can be used for confirmatory detection and identification of drought, WSMV+TriMV infection, ND and aphid stresses in wheat with ~ 100% accuracy. HPLC analyses of plant leaves revealed that WSMV+TriMV infection, ND and aphid stresses resulted in a significant decrease in the concentration of lutein, chlorophyll, and pheophytin. Similar changes in the intensity of carotenoid vibration (1185 cm^-1^) were also evident in the corresponding Raman spectra collected from wheat leaves. These results show that RS detects changes in the concentration of carotenoids that are taken place upon biotic and abiotic stresses. Furthermore, detailed analysis of changes in the concentration of lutein, chlorophyll, and pheophytin, as well as consideration of optical properties of these compounds, allows us to conclude that RS primarily detects changes in lutein in plant leaves.

## Data availability statement

The raw data supporting the conclusions of this article will be made available by the authors, without undue reservation.

## Author contributions

SH performed HPLC analyses of samples, analyzed data; performed chemometric analysis of data. VS acquired Raman spectra. BH grown plants, modelled stresses, collected samples. KG supervised the work, wrote the manuscript. DK supervised the work, wrote the manuscript. All authors contributed to the article and approved the submitted version.

## Funding

This study was supported by funds from Texas A&M AgriLife Research, Texas A&M University Governor’s University Research Initiative (GURI) grant program of (12-2016/M1700437).

## Conflict of interest

The authors declare that the research was conducted in the absence of any commercial or financial relationships that could be construed as a potential conflict of interest.

## Publisher’s note

All claims expressed in this article are solely those of the authors and do not necessarily represent those of their affiliated organizations, or those of the publisher, the editors and the reviewers. Any product that may be evaluated in this article, or claim that may be made by its manufacturer, is not guaranteed or endorsed by the publisher.
