# Supplementary material for: Confirmatory detection and identification of biotic and abiotic stresses in wheat using Raman spectroscopy
Source: Front Plant Sci. 2022 Oct 17;13:1035522. doi: 10.3389/fpls.2022.1035522 (PMC9618938; doi:10.3389/fpls.2022.1035522)
Supplement: Supplementary file 1 [file DataSheet_1.docx]

Confirmatory Detection and Identification of Biotic and Abiotic Stresses in Wheat Using Raman Spectroscopy

Samantha Higgins^1†^, Valerya Sereda^1†^, Benjamin Herron^2^, Kiran R. Gadhave^2,3*^ and Dmitry Kurouski^1*^

† Authors contributed equally to this work.

1. Department of Biochemistry and Biophysics, Texas A&M University, College Station, Texas 77843, United States

2. Texas A&M AgriLife Research, Amarillo, Texas 79106, United States

3. Department of Entomology, Texas A&M University, College Station, Texas 77843, United States

| Normalized peak intensities at the most prominent vibrational bands | | | | | | | | | |
| --- | --- | --- | --- | --- | --- | --- | --- | --- | --- |
|  | 748 cm^-1^ | 1002  cm^-1^ | 1155  cm^-1^ | 1185  cm^-1^ | 1217  cm^-1^ | 1285  cm^-1^ | 1326  cm^-1^ | 1525  cm^-1^ | 1602  cm^-1^ |
| Healthy | 0.073 | 0.082 | 0.229 | 0.122 | 0.069 | 0.036 | 0.070 | 0.306 | 0.028 |
| Drought | 0.065 | 0.074 | 0.222 | 0.113 | 0.061 | 0.034 | 0.064 | 0.302 | 0.027 |
| WSMV+TriMV | 0.062 | 0.071 | 0.196 | 0.102 | 0.058 | 0.033 | 0.065 | 0.279 | 0.029 |
| Nitrogen | 0.047 | 0.045 | 0.128 | 0.060 | 0.031 | 0.024 | 0.042 | 0.185 | 0.045 |
| Aphid | 0.042 | 0.060 | 0.177 | 0.050 | 0.023 | 0.026 | 0.034 | 0.284 | 0.035 |

**Supplementary Information**

**Table S1. Normalized peak intensities at the most prominent vibrational bands**

**748**

**
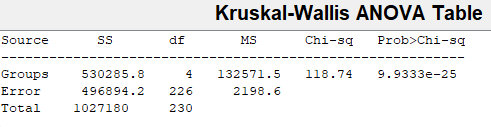
**

**1002**

**
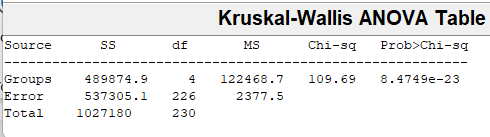
**

**1155**

**
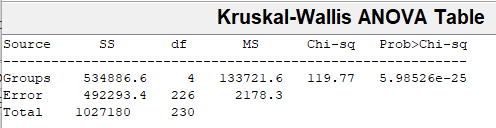
**

**1185**

**
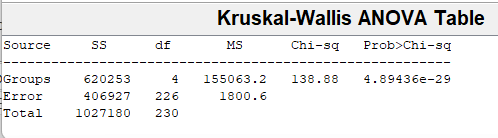
**

**1217**

**
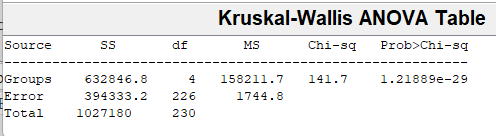
**

**1285**


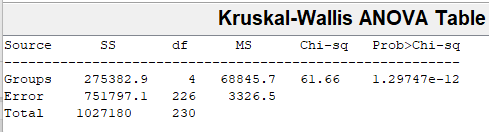

**1326**

**
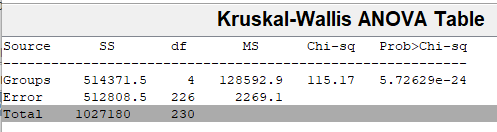
**

**1525**

**
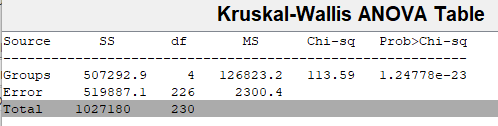
**

**1602**

**
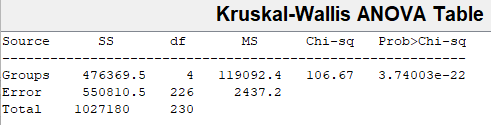
**

Figure S1. ANOVA of intensity of different vibrational bands in the acquired Raman spectra.
